# Transthoracic device closure of perimembranous ventricular septal defect via a small left intercostal incision in children

**DOI:** 10.3389/fcvm.2023.1221136

**Published:** 2023-08-21

**Authors:** Linfeng Xie, Guican Zhang, Jian He, Yanming Shen, Dongshan Liao, Liangwan Chen, Fan Xu

**Affiliations:** ^1^ Department of Cardiovascular Surgery, Fujian Medical University Union Hospital, Fuzhou, China; ^2^ Fujian Medical University, Fuzhou, China; ^3^ Fujian Provincial Center for Cardiovascular Medicine, Fuzhou, China

**Keywords:** congenital heart diseases, ventricular septal defect, transthoracic device closure, small incision, children

## Abstract

**Background:**

In children with perimembranous ventricular septal defect, surgical repair requires sternotomy and leaves unsightly scars, which can trigger long-term physical and psychological distress. However, transcatheter device closure is limited by vascular diameter, radiographic exposure, and expensive DSA equipment. We used an ultra-small left intercostal incision for transthoracic device closure to avoid the above problems and investigated its safety and feasibility by comparing it with surgical repair.

**Methods:**

This study enrolled 358 children with perimembranous ventricular septal defect. Among them, 152 patients were treated by surgical closure and 206 by transthoracic device closure via an ultra-small left intercostal incision. Perioperative clinical data and postoperative follow-up results were collected and analyzed retrospectively.

**Results:**

The success rate was similar (*P* = 0.265) in the two groups: 203/206 patients in the device group vs. 152/152 patients in the surgical group. The operative time, intensive care unit time, mechanical ventilation time, and postoperative hospital stay were significantly shorter in the device group than in the surgical group. Although the incision length of the device group (1.1 ± 0.2 cm) was significant shorter (*P* < 0.001) than that of the surgical group (6.7 ± 1.5 cm), there was no difference in hospitalization costs between the two groups (*P* = 0.099). Except for small residual shunt (16/206 vs. 3/152, *P* = 0.017), the incidence of complications in the device group was lower or equal to that in the surgical group, and all small residual shunt disappeared during follow-up. There was no thoracic deformity in the device group, compared with 11 cases in the surgery group during follow-up (*P* < 0.001).

**Conclusions:**

Transthoracic device closure via an ultra-small left intercostal incision under transesophageal echocardiography guidance is safe and feasible. With appropriate indications, it can be a suitable alternative to surgical closure for treating perimembranous ventricular septal defect in children.

## 1. Introduction

Ventricular septal defect (VSD) is one of the most common congenital heart diseases (CHD) and constitutes approximately 20% of all CHD, of which 80% is perimembranous ventricular septal defect (PmVSD) (1, 2). Asymptomatic patients with PmVSD can be treated when they are older, but those who suffer from congestive heart failure, failure to thrive, or progressive pulmonary hypertension need early medical treatment (3). Surgical repair is the standard treatment for PmVSD; however, thoracotomy and unsightly scar can trigger long-term physical and psychological distress. Transcatheter device closure avoids the above problems and achieves good clinical results, but its use in little children is limited by vascular diameter and radiographic exposure (4), and expensive digital subtraction angiography (DSA) equipment and much higher medical costs make its use in developing countries more challenging. Transthoracic minimally invasive device closure (TMIDC) does not mandate full sternotomy and cardiopulmonary bypass (CPB), without vascular diameter restriction and radiographic exposure, is considered suitable for children with PmVSD (5). Nevertheless, TMIDC still requires an incision of about 3–5 cm and partial sternotomy, which may cause obvious scars and even chest deformity (6). We used an ultra-small left intercostal incision to perform transthoracic device closure in children with PmVSD without opening the sternum for better cosmetic results. This technique is easy to learn, does not require expensive DSA equipment, and the costs are acceptable for developing countries.

This study aimed to investigate the safety and feasibility of transthoracic device closure of PmVSD via an ultra-small left intercostal incision in children by comparing it with surgical repair.

## 2. Materials and methods

The study involving human participants were reviewed and approved by the ethics committee of Fujian Medical University Union Hospital. Written informed consent for participation was not required for this study in accordance with the national legislation and the institutional requirements.

### 2.1. Patients

From January 2019 to December 2021, all 358 PmVSD patients treated at Fujian Medical University Union Hospital who met both inclusion and exclusion criteria were included in this study. Among them, 206 patients who underwent transthoracic device closure via an ultra-small left intercostal incision were assigned to the device group, and 152 patients who underwent surgical repair through a standard median sternotomy were assigned to the surgical group. Clinical data of the two groups derived from medical history inquiry, physical examination, electrocardiogram, chest radiograph, and echocardiogram during hospitalization were retrospectively collected and analyzed.

To ensure comparability, the inclusion and exclusion criteria were the same in both two groups. Patients with isolated PmVSD were enrolled if they had significant clinical symptoms (frequent respiratory infections, failure to thrive or repeated heart failure) and significant heart enlargement or progressive pulmonary hypertension. In patients with a PmVSD diameter of less than 5 mm, transthoracic echocardiography (TTE) should be performed at least twice with intervals of 6 months to ensure that the defects do not close spontaneously. The exclusion criteria were as follows: (1) large non-restrictive PmVSD; (2) aged less than 6 month; (3) PmVSD diameter less than 3 mm; (4) aortic valve prolapse or obvious aortic regurgitation; (5) a subaortic rim of the VSD less than 1 mm; (6) right to left shunt. Patient's guardian was informed of all available treatments in detail, and the treatment option was chosen according to their wishes.

### 2.2. Device and delivery system

**Figure 1:**
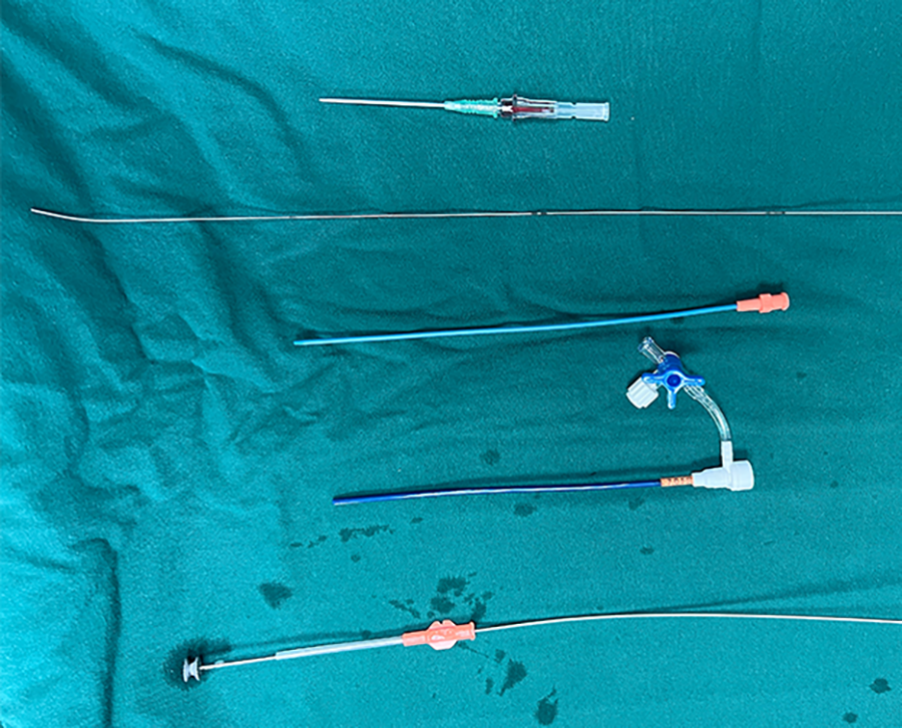
The delivery system and occluder.

The delivery system and occluder used in this study were domestic products (Shan Dong Visee Medical Apparatus, Wei Hai, China). The delivery system included a puncture needle, a guidewire, a dilator, a delivery sheath, and a loading sheath (Figure 1). Asymmetric and symmetric occluders are available, both of which are self-expandable, shape-memory functional, double-disc devices made from an alloy of nickel and titanium. On the asymmetric occluder, the left ventricular disc is eccentric, with the aortic side 1 mm wider than the waist and the apical side 5 mm wider than the waist with a platinum marker. On the symmetric occluder, both discs are circular and are 2 mm wider than the waist. The occluder is sized based on the waist diameter ranging from 4 to 18 mm.

### 2.3. Transesophageal echocardiography and device selection

A Philips IE33 echocardiography instrument with a pediatric esophageal ultrasound probe S7-3T was used in this study. After general anesthesia and intubation, transesophageal echocardiography (TEE) was performed to evaluate the location, size, and adjacent structures of PmVSD, especially the margins adjacent to the aortic valve. An asymmetric occluder was used if the distance between the upper margin of PmVSD and the lower margin of the aortic valve was less than 2 mm; otherwise, a symmetric occluder was used. The occluder size was based on the largest diameter of the PmVSD, with the waist size 1–2 mm larger than the diameter of the defect.

### 2.4. Transthoracic device closure

**Figure 2:**
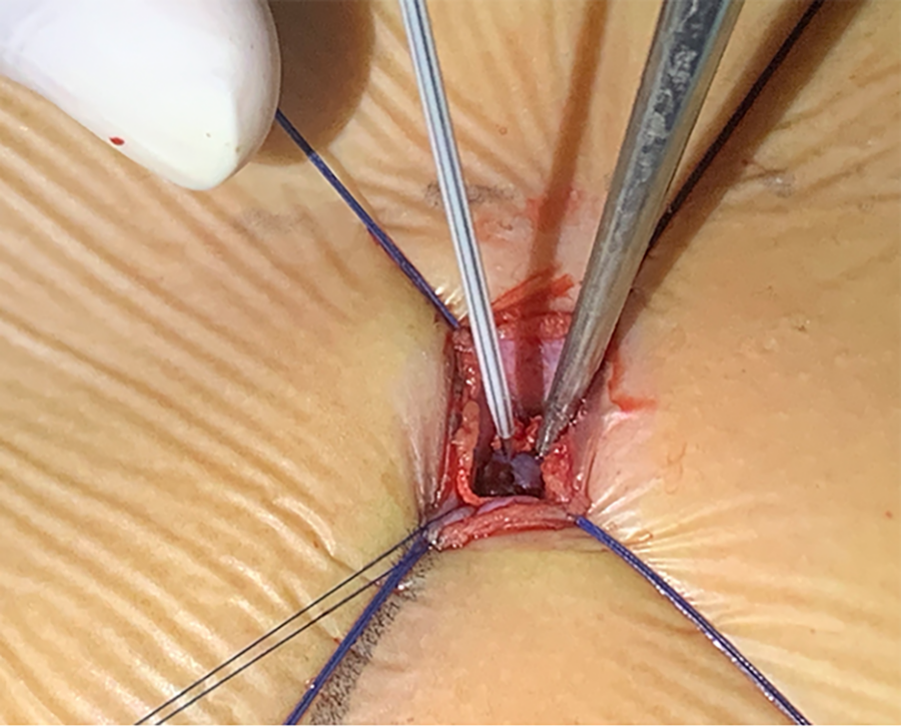
Lift the purse string suture and puncture the right ventricle.

**Figure 3:**
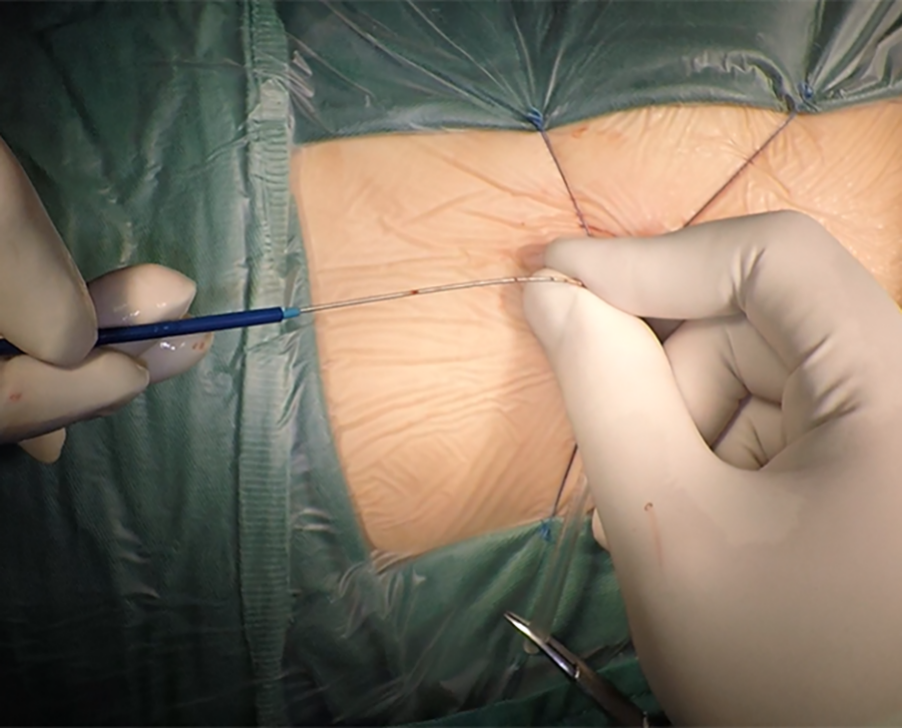
The delivery sheath was inserted along the guidewire to established delivery system.

**Figure 4:**
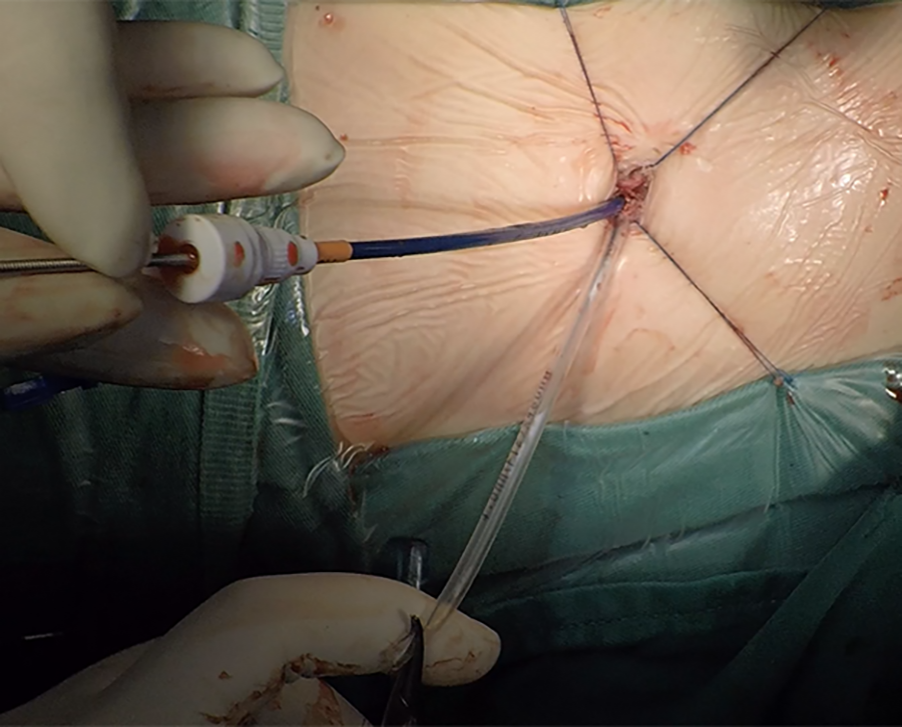
Deployed of the occluder to ventricular septal defect via the delivery system.

**Figure 5:**
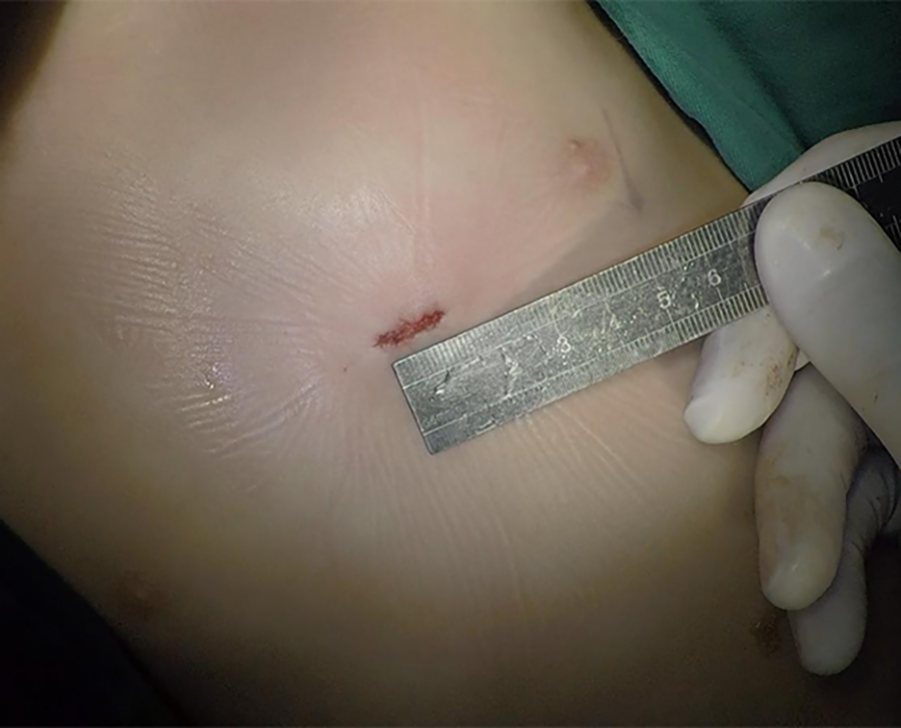
One centimeter ultra-small left intercostal incision without a drainage tube.

The patient was placed in the left slope decubitus position, and TTE was repeated to determine the position of the trans intercostal incision according to the location of the PmVSD and the direction of blood flow. The incision was made between the left third to fifth intercostal space, of which the fourth intercostal space is the most common, with a length of about 1 cm and at least 1 cm from the sternum. Blunt dissection of subcutaneous tissue and intercostal muscle was performed to expose the naked area of the pericardium. The pericardium was opened and suspended to expose the right ventricle. Under TEE guidance, the right ventricular surface was lightly pressed with tweezers, and the point parallel to the direction of blood flow across the PmVSD with the shortest distance was selected as the final puncture point. Heparin (1 mg/kg) was administered to maintain an activated clotting time of >250 s. A purse string was sutured at the puncture site, the puncture needle was inserted into the right ventricle through the purse string (Figure 2). A guidewire passed through the puncture needle entered the left ventricle through PmVSD under TEE guidance. The delivery sheath was inserted along the guidewire (Figure 3), ensuring that its tip entered the left ventricle at a safe distance from the aortic valve. The occluder was loaded into the loading sheath. The guidewire and the inner core of the delivery sheath were removed, and the loading sheath was connected to the end of the delivery sheath. The delivery cable was pushed forward to deliver the occluder to the left ventricle along the delivery sheath (Figure 4). With TEE guidance, we released the left ventricular disc to ensure a safe distance from the aortic valve. If an asymmetrical occluder was used, it was rotated slightly until the platinum marker on the apical side pointed downwards, and then the waist and the right ventricular disc were deployed. The occluder was released if there was no significant residual shunt, moderate to severe valve regurgitation or arrhythmia. The sheath and the delivery cable were withdrawn, and the suture was tied snugly. The chest was closed routinely without a drainage tube (Figure 5). Oral aspirin (3 mg/kg/day) was given for 3 months as an anticoagulant. VSD closure is considered successful if the following conditions are met: (1) no residual shunt or no significant residual shunt (<2 mm in width and 2 m/s in velocity), (2) no new onset of moderate-to-severe valve regurgitation. (3) no severe atrioventricular block.

### 2.5. Surgical closure

Surgical closure was performed using a standard median sternotomy approach under CPB, and autologous pericardial patches were used in all patients.

### 2.6. Statistical analysis

The data were analyzed by SPSS version 26.0 (IBM, Armonk, NY). As a retrospective study, propensity score matching (PSM) was used to reduce the effects of selection bias and potential confounding. Patients in the device group were matched with those in surgical group used nearest-neighbor matching (1:1) with a caliper value of 0.02 without replacement. Matching factors included age, sex, weight, VSD size and pulmonary arterial pressure. All continuous variables were confirmed as normal distribution by Kolmogorov–Smirnov test, and presented as mean ± standard deviation. Therefore, comparisons of continuous variables between groups used the independent samples *t*-test. Categorical variables were expressed by numbers and percentages (*n*, %). Comparisons of categorical variables between groups used the Chi-square test or, if necessary, Fisher's exact test. A *P*-value <0.05 was considered statistical significance.

## 3. Results

After PSM, there were 150 patients in each of the two groups were matched. There were no significant differences in comparisons of clinic data between the two groups before and after PSM.

Table 1 shows no significant differences in the preoperative clinical data between the two groups, indicating that the patients in the two groups are homogeneous and comparable.

**Table 1:** Comparison of preoperative clinical data between two groups.

	All patients			Propensity score matching patients		
	Device group	Surgical group	*P*-value	Device group	Surgical group	*P*-value
Number of patients	206	152	–	150	150	–
Sex (male/female)	76/130	65/87	0.275	64/86	64/86	1.000
Age (year)	1.72 ± 0.79	1.59 ± 0.70	0.136	1.64 ± 0.78	1.60 ± 0.71	0.620
Weight (kg)	10.4 ± 3.4	9.9 ± 3.1	0.110	9.8 ± 3.1	9.9 ± 3.1	0.910
VSD size (mm)	5.1 ± 1.6	5.3 ± 1.9	0.191	5.3 ± 1.7	5.4 ± 1.9	0.877
Pulmonary arterial pressure (TTE, mmHg)	41.8 ± 13.8	43.4 ± 12.9	0.279	43.5 ± 13.8	43.3 ± 13.0	0.904
Normal (*n*, %)	22 (10.7%)	11 (7.2%)	–	15 (10.0%)	11 (7.3%)	–
Mild pulmonary hypertension (*n*, %)	133 (64.6%)	99 (65.1%)	0.666	94 (62.3%)	97 (64.7%)	0.683
Moderate pulmonary hypertension (*n*, %)	42 (20.4%)	36 (23.7%)	–	32 (21.3%)	36 (24.0%)	–
Severe pulmonary hypertension (*n*, %)	9 (4.4%)	6 (4.0%)	–	9 (6.0%)	6 (4.0%)	–
Cardiothoracic ratio	0.57 ± 0.09	0.59 ± 0.10	0.109	0.58 ± 0.10	0.59 ± 0.10	0.216
Heart failure (*n*, %)	19 (9.2%)	12 (7.9%)	0.659	19 (12.7%)	12 (8.0%)	0.184

Kg, kilogram; mm, millimeter; TTE, transthoracic echocardiography; mmHg, millimeters of mercury.

There was no death in either group. After PSM, closure was successful in 147 of 150 patients (98.0%) in the device group, with 125 symmetric occluders and 22 asymmetric occluders used. Closure failed in the remaining 3 patients because of 1 case of complete atrioventricular block (CAVB) after the surgery, 1 case of a significant residual shunt, and 1 case of failure in establishing a delivery system. The case of CAVB developed on the third postoperative day and was transferred to surgical repair. The occluder was removed and temporary pacemaker was installed, the sinus rhythm was converted after glucocorticoid treatment. One patient experienced a significant residual shunt after the complete release of an asymmetric occluder. The guidewire could not be passed through the PmVSD to establish the delivery system for another patient. These two patients were transferred to surgical repair. All 150 patients in the surgical group were successfully operated on, with no significant difference in success rate between the two groups (*P* = 0.247).

Table 2 shows significant differences in perioperative clinical data between the two groups. The operative time, intensive care unit (ICU) time, mechanical ventilation time, and postoperative hospital stay in the device group were significantly shorter than those in the surgical group (All *P* < 0.001), no matter before or after the PSM. The device group had a shorter incision (1.2 ± 0.2 cm vs. 6.7 ± 1.5 cm, *P* < 0.001) with no drainage tube or blood transfusion requirements (0/150 vs. 17/150, *P* < 0.001). However, there was no significant difference between the two groups for hospitalization costs (36,806.6 ± 5,205.9 vs. 36,166.4 ± 4,652.1, *P* = 0.262).

**Table 2:** Comparison of perioperative clinical data between two groups.

	All patients			Propensity score matching patients		
	Device group	Surgical group	*P*-value	Device group	Surgical group	*P*-value
Number of patients	206	152	–	150	150	–
Procedure success (*n*, %)	203 (98.5%)	152 (100%)	0.265	147 (98.0%)	150 (100%)	0.247
Operative time (min)	39.6 ± 14.7	124.6 ± 20.5	<0.001	40.9 ± 15.1	125.0 ± 20.4	<0.001
Cardiopulmonary bypass time (min)	–	65.2 ± 18.3	–	–	65.4 ± 18.3	–
Aortic cross-clamp time (min)	–	34.3 ± 9.2	–	–	34.2 ± 9.3	–
Mechanical ventilation time (h)	2.7 ± 0.7	12.1 ± 3.8	<0.001	2.7 ± 0.7	12.1 ± 3.8	<0.001
Intensive care unit time (h)	7.5 ± 2.0	21.1 ± 5.4	<0.001	7.4 ± 2.0	21.0 ± 5.4	<0.001
Postoperative hospital stays (h)	54.5 ± 21.7	148.9 ± 44.2	<0.001	55.4 ± 22.5	149.0 ± 44.3	<0.001
Drainage (ml)	–	51.6 ± 21.0	–	–	51.5 ± 21.0	–
Blood transfusion (*n*, %)	0 (0%)	17 (11.2%)	<0.001	0 (0%)	17 (11.2%)	<0.001
Incision length (cm)	1.1 ± 0.2	6.7 ± 1.5	<0.001	1.2 ± 0.2	6.7 ± 1.5	<0.001
Hospitalization costs (RMB)	37,103.3 ± 5,061.7	36,080.7 ± 4,685.7	0.099	36,806.6 ± 5,205.9	36,166.4 ± 4,652.1	0.262

RMB, Renminbi (unit of Chinese currency).

Table 3 shows the comparison of postoperative complications between the two groups. There was no new-onset aortic regurgitation, hemolysis, or occluder dislodgement in both groups. The incidence of small residual shunts (less than 2 mm) was higher in the device group than in the surgical group (11/150 vs. 3/150, *P* = 0.029), but the incidence of pulmonary infection (5/150 vs. 15/150, *P* = 0.021) and anemia (4/150 vs. 22/150, *P* < 0.001) was significantly lower in the device group than in the surgical group. Transient arrhythmia occurred in 12 cases in the device group and 13 cases in the surgical group (*P* = 0.835), and all recovered after antiarrhythmic therapy. Although there was no statistical difference in the incidence of pericardial effusion, pleural effusion, and pneumothorax between the two groups, there was no corresponding case in the device group.

**Table 3:** Comparison of postoperative complications between two groups before discharge.

	All patients			Propensity score matching patients		
	Device group	Surgical group	*P*-value	Device group	Surgical group	*P*-value
Number of patients	206	152	–	150	150	–
Occluder dislodgement (*n*, %)	0 (0%)	–	–	0 (0%)	–	–
Small residual shunt (<2 mm) at discharge (*n*, %)	16 (7.8%)	3 (2.0%)	0.017	11 (7.3%)	3 (2.0%)	0.029
Significant residual shunt requiring reoperation (*n*, %)	1 (0.5%)	0 (0%)	1.000	1 (0.7%)	0 (0%)	1.000
Complete atrioventricular block (*n*, %)	1 (0.5%)	0 (0%)	0.510	1 (0.7%)	0 (0%)	1.000
Transient arrhythmia (*n*, %)	15 (7.3%)	13 (8.6%)	0.694	12 (8.0%)	13 (8.7%)	0.835
New-onset aortic insufficiency (*n*, %)	0 (0%)	0 (0%)	–	0 (0%)	0 (0%)	–
New-onset tricuspid insufficiency (*n*, %)	13 (6.3%)	8 (5.3%)	0.821	10 (6.7%)	8 (5.3%)	0.627
Hemolysis (*n*, %)	0 (0%)	0 (0%)	–	0 (0%)	0 (0%)	–
Anemia (*n*, %)	5 (2.4%)	23 (15.1%)	<0.001	4 (2.7%)	22 (15.1%)	<0.001
Pulmonary infection (*n*, %)	6 (3.0%)	15 (9.9%)	0.007	5 (3.3%)	15 (10%)	0.021
Pericardial effusion (*n*, %)	0 (0%)	3 (2.0%)	0.076	0 (0%)	3 (2.0%)	0.247
Pneumothorax (*n*, %)	0 (0%)	1 (0.7%)	0.425	0 (0%)	1 (0.7%)	1.000
Pleural effusion (*n*, %)	0 (0%)	2 (1.3%)	0.180	0 (0%)	2 (1.3%)	0.498

### 3.1. Follow-up

All patients received an electrocardiogram, TTE, and physical examination 3 and 12 months after surgery and then as per need. The median follow-up period was 13.6 months. Table 4 shows the comparison of clinical data during follow-up. During the follow-up period, there was no occluder dislodgement, late-onset CAVB, late-onset aortic insufficiency or tricuspid insufficiency. The new-onset tricuspid insufficiency after surgery disappeared or remained unchanged in the two groups. Three months after surgery, there were only 2 cases with small residual shunts in the device group and 1 case in the surgical group (*P* = 1.000). All small residual shunts disappeared 12 months after surgery. Eleven patients in the surgical group were found to have thoracic deformities, including 8 cases with pectus carinatum and 3 cases with funnel chest, while none were found in the device group (*P* < 0.001).

**Table 4:** Comparison of clinical data between the two groups during follow-up.

	All patients			Propensity score matching patients		
	Device group	Surgical group	*P*-value	Device group	Surgical group	*P*-value
Number of patients	206	152	–	150	150	–
Occluder dislodgement (*n*, %)	0 (0%)	–	–	0 (0%)	–	–
Late-onset complete atrioventricular block (*n*, %)	0 (0%)	0 (0%)	–	0 (0%)	0 (0%)	–
Late-onset aortic insufficiency (*n*, %)	0 (0%)	0 (0%)	–	0 (0%)	0 (0%)	–
Late-onset tricuspid insufficiency (*n*, %)	0 (0%)	0 (0%)	–	0 (0%)	0 (0%)	–
Small residual shunt at 3th month after surgery (*n*, %)	3 (1.5%)	1 (0.7%)	0.640	2 (1.3%)	1 (0.7%)	1.000
Small residual shunt at 12th month after surgery (*n*, %)	0 (0%)	0 (0%)	–	0 (0%)	0 (0%)	–
Thoracic deformity (*n*, %)	0 (0%)	11 (7.2%)	<0.001	0 (0%)	11 (7.3%)	<0.001

## 4. Discussion

TMIDC has been used in clinical practice and achieved satisfactory clinical effect over the last decade (7, 8). It provided another minimally invasive option for children with PmVSD, especially those who cannot receive transcatheter device closure or tolerate CPB (9). Sawing the lower sternum into the chest is the earliest and most widely used method. Although the technique is less traumatic than surgical repair, it leads to obvious scars and even sternal deformity in the middle of the chest, increasing the psychological burden in the future (10), especially in children and female patients. If the closure fails, PmVSD can only be repaired by median sternotomy under CPB, we used an ultra-small left intercostal incision to perform transthoracic device closure in children with PmVSD and avoid the above problems. As surgical repair with median sternotomy is the standard method for treating PmVSD, this study evaluated the safety and feasibility of transthoracic device closure of PmVSD via an ultra-small left intercostal incision by comparing it with surgical repair.

With advancements in modern surgical techniques, the aim is to achieve the effectiveness of surgery and cosmetic improvement (11). However, surgeons face the challenges of obtaining optimal esthetics and clear surgical vision. As surgical repair of PmVSD requires adequate vision to establish CPB, the incision is usually about 6–10 cm and leaves a significant postoperative scar (12). Meanwhile, only the puncture point on the right ventricle needs exposure during TMIDC. We defined the border of the left lung on a chest radiograph and determined the position of PmVSD and the direction of trans PmVSD blood flow using TTE to select the most appropriate location for intercostal incision. Thus, the incision length was reduced to nearly 1 cm. This approach does not require sternotomy, and pericardial suspension avoids rib injury; therefore, no chest deformity occurred in the device group. Since the procedure involved minimal trauma and almost no intraoperative bleeding, a drainage tube was not needed, avoiding additional scars. Moreover, the incision could be concealed after breast development, increasing acceptability among female patients.

Generally, a small incision can limit the scope of the operation and increase surgical difficulty. However, this study showed no significant difference in the success rate between the two groups. Only one of 206 patients in the device group showed failure in establishing the delivery system. Accurate assessment of the position of PmVSD and the direction of trans PmVSD blood flow by TTE to determine the optimal incision position is very important for improving success rates. Usually, after opening the pericardium via an intercostal incision, the appropriate puncture point is located near the incision. When establishing the delivery system during the operation, the direction of the guidewire is often adjusted laterally to pass through the PmVSD, and the intercostal incision is just transverse, which will not limit the operation.

In addition to esthetics, the safety of surgery is also important. Mortality, incidence, and severity of complications are important indicators to evaluate the safety of surgery. This study showed no deaths in both groups. Except for small residual shunt, the complication rate of the device group was lower or equal to that of the surgery group. The gap between the occluder and PmVSD was the main reason for the higher incidence of small residual shunts in the device group (13). After full deployment of the occluder and final coverage of the occlude by proliferating endothelial cells, the small residual shunt usually disappears within weeks to months after surgery, confirmed by our follow-up results. These data suggested that although our approach used a smaller incision, it did not compromise safety.

**Figure 6:**
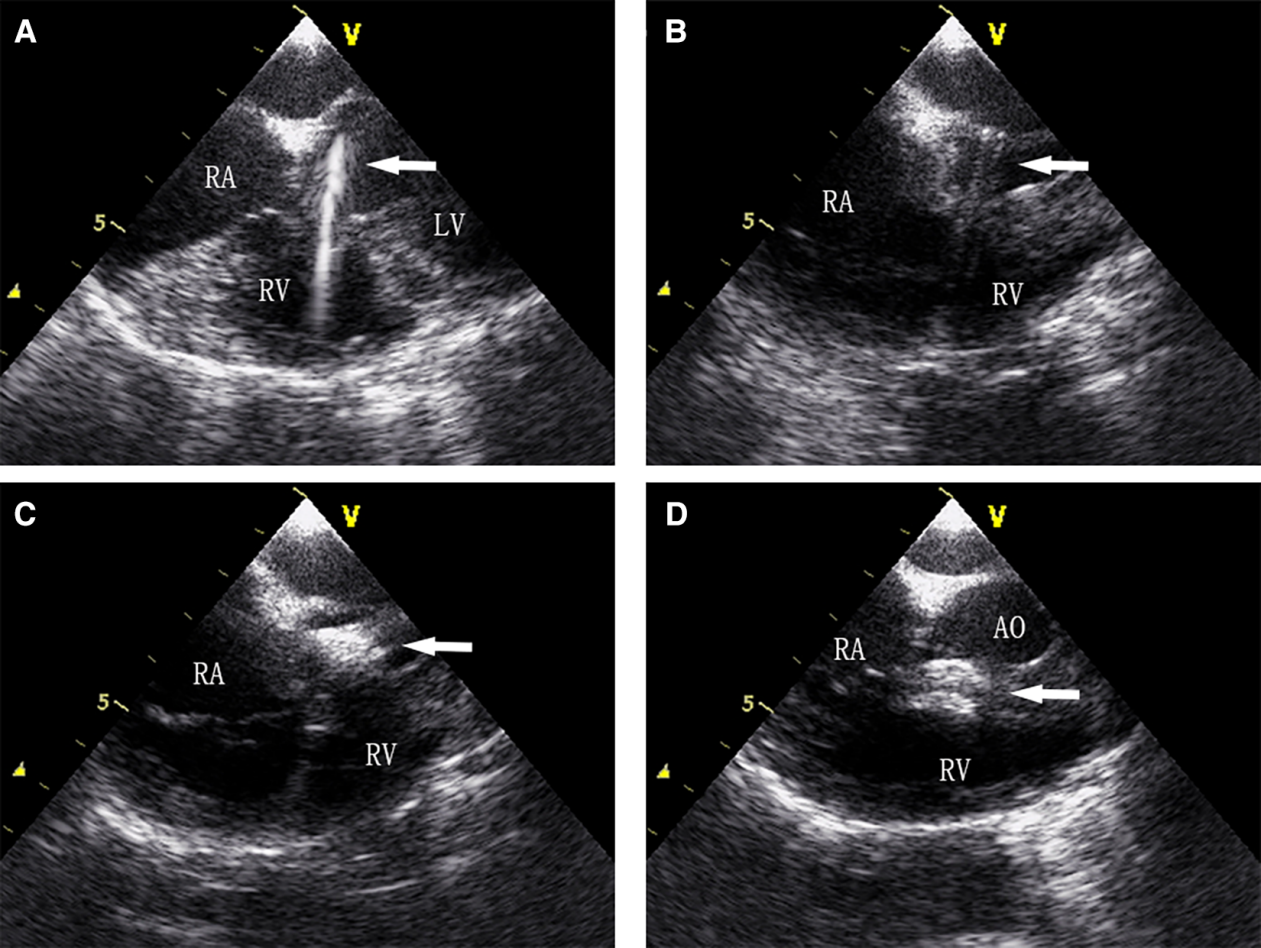
(**A**) Guidewire was sent to the left ventricle through the PmVSD, (**B**) delivery systems were established, (**C**) the left ventricular disc is deployed, (**D**) both discs are deployed to occlude the PmVSD.

Aortic insufficiency and CAVB are reasons that transcatheter device closure of PmVSD has not been widely adopted. In this study, the incidence of aortic insufficiency and CAVB was low, with only one patient having CAVB and none having aortic insufficiency. We believe an accurate assessment of the location, size, morphology, and adjacent structures of PmVSD is essential to reduce the occurrence of aortic insufficiency. If the distance between the upper margin of PmVSD and the lower margin of the aortic valve is less than 2 mm, an asymmetric occluder should be selected to avoid the impact on the aortic valve. If the distance is less than 1 mm, PmVSD is combined with aortic valve prolapse, or the tricuspid valve is involved in the formation of membranous aneurysms, surgical repair is a better choice. Besides, TEE can display important structures around PmVSD in real-time in a beating heart, such as aortic, tricuspid, and mitral valves, to avoid damage (Figure 6). Even if regurgitation occurs, it can be detected and adjusted in time. Therefore, there was no new-onset aortic regurgitation in the device group in this study. Arrhythmia was the most common complication in both groups, with CAVB being the most serious complication. It was reported the following reasons for CAVB induced by device closure: (1) the occluder compressing the conduction system; (2) adjacent tissue edema involving the conduction system; (3) inflammatory reaction or scar formation of the conduction system (14, 15). Butera believed that the progressive deployment of the occluder was the cause of CAVB (16). Either way, the core of the problem is the impact of surgical procedures and occluder on the conduction system. Unlike transcatheter device closure, transthoracic device closure via an ultra-small left intercostal incision resulted in a shorter entrance route and fewer turns of the delivery sheath inside the heart, making it easier to approach the PmVSD perpendicularly under the real-time guidance of TEE and significantly reduced the impact of surgical procedures on the conduction system (17). To improve the safety, a device protective suture of 3–0 polypropylene was passed through the mesh of the right ventricular disc during the surgery. Due to the use of the protective suture, the occluder can be retrieved after being released from the delivery sheath, allowing the occluder to be fully deployed to simulate its effect on the conduction system in the postoperative physiological state and to assess the appropriate size and location of the occluder. Moreover, selecting the smallest possible occluder to minimize its effect on the conduction system also helps reduce the incidence of CAVB (18). Even so, we believe that surgical repair is still irreplaceable in some situations.

The right ventricular surface of PmVSD is adjacent to the tricuspid valve, so either surgical repair or device closure may induce tricuspid insufficiency. Chordae tendineae or valve injury by catheter and occluder are considered as risk factors for tricuspid insufficiency during device closure procedure (19). In this study, there was no difference in the incidence of tricuspid insufficiency between the two groups, which was similar to other studies (20, 21). During the follow-up, all patients with mild new-onset tricuspid insufficiency recovered well without any progress and no further intervention is needed. It may be related to the memory alloy remodeling process can gradually reduce tricuspid regurgitation. In general, mild new-onset tricuspid insufficiency can be just observed because it usually disappears or shows a decrease in its severity during the follow-up. However, for moderate to severe new-onset tricuspid insufficiency, switch to surgical repair maybe a better choice.

Pneumothorax and the injury to the left internal thoracic artery are complications that require vigilance when planning the left intercostal incision. Chest radiographs can determine the border of the left lung to avoid pneumothorax. During the operation, we used the left slope decubitus position to shift the heart to the left so that the intercostal incisions could be as far from the sternum as possible. In this study, all incisions were at least 1 cm from the left margin of the sternum, and the left internal thoracic artery showed no damage. TTE can detect the internal thoracic artery blood flow and help avoid damage in some children.

As cost is an important factor influencing the choice of the operation technique in developing countries, we selected a domestic device to minimize costs. Occluder costs were higher in the device group, but the costs for ICU and hospitalization were lower than in the surgical group; thus, the average total cost was similar between the two groups. Furthermore, transthoracic device closure via an ultra-small left intercostal incision does not require expensive DSA equipment and is easier to perform in primary hospitals. However, the study was conducted in a developing country, and the cost-benefit calculations may differ in developed countries.

This study has some limitations. First, this was a single-center retrospective study, a larger, prospective randomized controlled study is needed to confirm the results. Second, a long-term follow-up was necessary to evaluate the following results. Thirdly, the comparison of car growing and psychological assessment were not conducted.

## 5. Conclusion

Transthoracic device closure via an ultra-small left intercostal incision under real-time TEE guidance is safe and feasible. With an appropriate indication, it can be a suitable alternative to surgical closure for treating PmVSD in children.

## Data Availability

The original contributions presented in the study are included in the article/Supplementary Material, further inquiries can be directed to the corresponding author.
